# Machine learning–driven identification and experimental validation of key biomarkers in the bile acid metabolic pathway associated with ulcerative colitis

**DOI:** 10.3389/fimmu.2026.1873644

**Published:** 2026-08-24

**Authors:** Yuqing Wu, Danyang Gu, Jin Liu, Yongbing Yang, Yaman Wang, Yangjing Wang, Yuan Mu, Ruihong Sun, Ben Huang

**Affiliations:** 1 Department of Medical Laboratory, The First Affiliated Hospital of Nanjing Medical University, Nanjing, Jiangsu, China; 2 Department of Geriatric Gastroenterology, The First Affiliated Hospital of Nanjing Medical University, Nanjing, Jiangsu, China; 3 Department of Oncology, Suqian First Hospital, Suqian, Jiangsu, China; 4 Department of Eye-X Research Institute, Bengbu Medical University, Bengbu, Anhui, China; 5 The First School of Clinical Medicine, Nanjing Medical University, Nanjing, Jiangsu, China

**Keywords:** bile acid metabolism, immune cell analysis, machine learning, single cell analysis, ulcerative colitis, WGCNA

## Abstract

**Background:**

Bile acids are shown to participate in inflammatory responses. This study was designed to investigate the functions of bile acid metabolism-associated genes (BAMGs) in ulcerative colitis (UC), identify the potential biomarkers based on eleven machine learning algorithms.

**Methods:**

Seven independent UC transcriptomic datasets were retrieved from the GEO database. Differentially expressed genes, weighted gene co-expression network analysis (WGCNA), and multiple machine learning algorithms were integrated to identify key BAMGs. Subsequently, enrichment analysis, immune cell analysis and single cell analysis were performed to explore the biological functions and immunological characteristics. The dextran sulfate sodium (DSS) induced colitis model in mice was then established and validated the results through western blot and immunohistochemical (IHC) analysis. In addition, peripheral blood samples were collected from UC patients for the detection of feature gene expression by quantitative real-time PCR (RT-qPCR).

**Results:**

Through integrative analysis, three feature BAMGs (*CH25H*, *SLC23A1* and *PHYH*) were identified. Unsupervised clustering based on the three-gene signature stratified UC patients into two distinct subgroups exhibiting divergent immune status. In DSS-treated mice, western blot and IHC confirmed significantly reduced SLC23A1 and PHYH protein levels and elevated CH25H protein expression in colonic tissues. RT-qPCR analysis of PBMCs from UC patients showed consistent gene expression. Immune cell analysis showed obvious association between the key BAMGs and inflammatory cells including naïve B cells, neutrophils, monocytes, CD8 T cells, and macrophages. Single-cell analysis revealed that the three feature genes were differentially expressed across T- and B-cell subsets, indicating their potential involvement in UC.

**Conclusion:**

This study identified a novel of BAMGs and preliminary revealed their interaction with immune cells in the development of UC. Downregulation of *SLC23A1* and *PHYH* and upregulation of *CH25H* may contribute to UC pathogenesis and represent potential biomarkers.

## Introduction

Ulcerative colitis (UC) is a chronic, multifactorial inflammatory disease characterized by persistent abnormal immune responses in the intestine. In recent years, the incidence of UC has gradually increased (1). Alongside typical symptoms such as abdominal pain, diarrhea and rectal bleeding, patients may also experience extraintestinal manifestations including arthritis and oral ulcers, which significantly impact patients’ quality of life. Furthermore, long-term presence of UC is closely associated with an increased risk of developing colon cancer (2, 3). In clinical practice, the diagnosis of UC primarily relies on clinical manifestations, laboratory tests, imaging examinations, and colonoscopy. Among the methods, colonoscopy serves as the cornerstone for diagnosing UC (4). While colonoscopy provides an intuitive diagnosis, its invasive testing may be limited by patient cooperation, especially for recurrent patients, it is not suitable for frequent use. Moreover, some patients exhibit atypical symptoms which can pose challenges. Therefore, searching for key biomarkers associated with the progression of UC is imperative for understanding the disease.

Bile acids, which are synthesized and catabolized in the liver, play an important role in regulating lipid, glucose, and energy metabolism. Studies have shown that dysregulation of bile acid homeostasis has been implicated in metabolic diseases, such as type 2 diabetes mellitus and nonalcoholic fatty liver disease (5). Therefore, bile acid signaling pathways have emerged as attractive therapeutic targets for the treatment of metabolic disorders (6). Specifically, Hao et al. (7) reported that gentiopicroside could reduce pathological bile acid accumulation in both serum and hepatic tissue, alleviate the inflammatory cell infiltration by regulating the expression of bile acid metabolism-related genes, such as *NTCP*, *MDR1* and *BSEP*. For patients with inflammatory bowel disease (IBD), abnormal bile acid metabolism has also been observed (8, 9). Research have suggested that bile acids could exert immunomodulatory effects by interacting with immune cells in IBD (10). After being released from the gallbladder and completion of lipolytic actions, bile acids traverse the digestive tract where they undergo modification by intestinal bacteria into immunomodulatory molecules. These modified bile acids subsequently activate T cells, regulating immune responses by either suppressing or promoting inflammation (10). Furthermore, experiments by Dennis Kasper et al. (11) have demonstrated that gut microbes and diet can collaboratively modify bile acids, thereby influencing the level of colonic Treg cell in mice, which affects the development of autoimmune diseases such as IBD. Together, these studies have identified a potential therapeutic approach for regulating intestinal inflammation involving bile acid modulation. Based on the evidence, we hypothesized that genes involved in the bile acid metabolism pathway could play important roles in the development of UC.

Machine learning, a core subfield of artificial intelligence, enables accurate prediction from large-scale, high-dimensional biomedical data and exhibits superior discriminative power and generalizability in disease classification, risk stratification, and treatment response prediction (12). Consequently, integrating data mining with machine learning–driven analytical frameworks has emerged as a robust strategy to enhance analytical precision, identify novel candidate biomarkers, and elucidate underlying disease mechanisms (12, 13). In the present analysis, we utilized the GSE92415 dataset from GEO database to systematically investigate the expression differences and immunological characteristics of bile acid metabolism-associated genes (BAMGs) between normal and UC samples. Subsequently, weighted gene co-expression network (WGCNA) analysis and 11 machine learning algorithms: Lasso regression analysis, Boruta analysis, Recursive Feature Elimination (RFE); Decision Trees (DT); Gradient Boosting Machine (GBM); General Linear Model (GLM); K-Nearest Neighbors (KNN); Neural Network (NNET); Random Forest (RF); Support Vector Machine (SVM) and eXtreme Gradient Boosting (XGB) were applied to identify the key BAMGs. The performance of the feature gene was verified using ROC analysis in three independent external independent UC tissue datasets (GSE87466, GSE87473, GSE107499) and three independent blood datasets (GSE126124, GSE119600 and GSE3365). Finally, using clinical blood samples and UC mouse model experiment, we successfully verified the expression of feature genes, demonstrating the candidate gene can serve as potential biomarkers for UC.

## Methods

### Data collection and processing

In this study, we downloaded seven UC datasets (GSE92415, GSE87466, GSE87473, GSE107499, GSE126124, GSE119600 and GSE3365) from GEO dataset (https://www.ncbi.nlm.nih.gov/geo/). The Details of the datasets were shown in **Supplementary Material**, **Supplementary Table 1**. The GSE126124, GSE119600, GSE3365 dataset contained peripheral blood sequencing RNA-seq data on UC patients, the other datasets were information based on the colonic mucosa. In the following analysis, we selected GSE92415 as the primary cohort due to its larger sample size of UC cases (n = 162). To avoid batch effects and platform-specific technical artifacts, the six additional RNA-seq GEO datasets were analyzed independently—rather than merged into a single cross-platform expression matrix—for downstream discovery analyses. The original gene expression matrix was processed using R version 4.4.3. Based on the platforms’ annotation information, the probes were converted to gene symbols. The log2 transformation and normalization were performed using “normalizeBetweenArrays” function from the “limma” package. Probes matching multiple genes were removed out and the average expression values were calculated as the final expression value.

**Figure 1:**
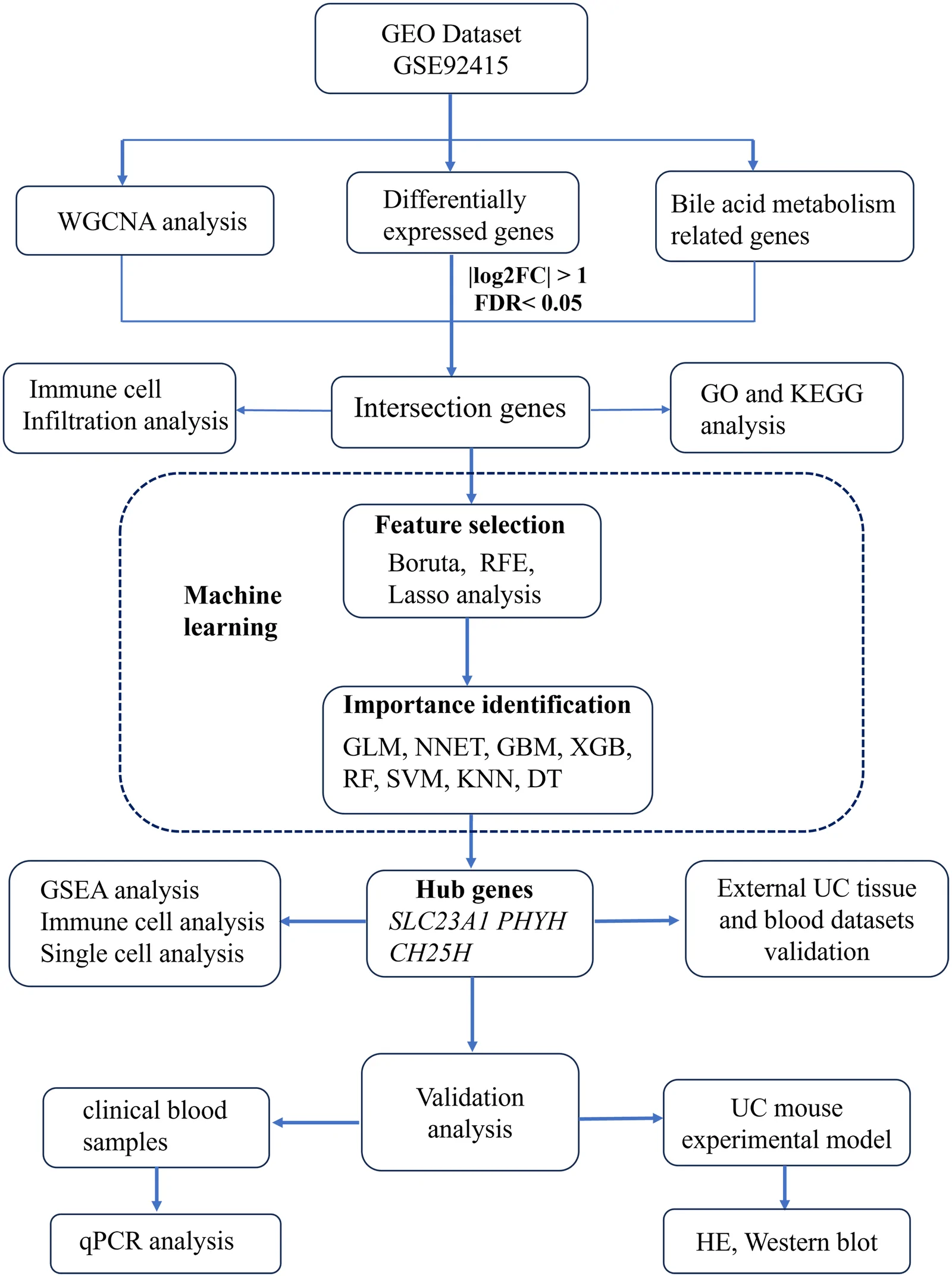
Flowchart of this study.

In addition, the bile acid metabolism related genes were obtained from the gene set enrichment analysis (GSEA) official website by the key word “bile acid metabolism” (https://www.gsea-msigdb.org/gsea/index.jsp). In total, 112 relevant genes were enrolled (**Supplementary Material**, **Supplementary Table 2****).** The overall workflow of this study was illustrated in **Figure 1**.

### Identification of differentially expressed BAMGs

The differentially expressed genes (DEGs) between UC and control samples were analyzed using “limma” package in R 4.4.3 software. DEGs was screened with the criteria: log2 fold change |log2FC| > 1 and adjust *P* < 0.05. The DEGs were visualized by using “pheatmap” and “ggplot2” packages. The “venndiagram” package was used to identify intersection gene between bile acid metabolism genes and DEGs.

### Functional enrichment analysis for BAMGs

To explore the biological and molecular pathways involved in BAMGs, the gene ontology (GO) and Kyoto Encyclopedia of Genes and Genomes (KEGG) enrichment analyses were performed by “clusterProfiler” package. In the subsequent analysis, we performed single-gene GSEA analysis by GSEA software (version 4.4.0). Samples were divided into high-expression and low-expression groups according to the median expression level of the target gene. Gene expression profiles and phenotypic grouping were analyzed with default parameters. The number of permutations was set to 1000. The “c2.cp.kegg_medicus.v2025.1.Hs.symbols.gmt” subset was used as the reference gene set and gene sets were set to a minimum size of 10 and a maximum size of 500. Gene sets with adjusted *P* < 0.05 were considered as significantly enriched. The “ggplot2” package was used for visualization.

### Analysis of immune cell infiltration

In this study, the CIBERSORT algorithm was employed to assess the immune cell infiltration within the microenvironment of UC and normal samples. The CIBERSORT algorithm employs linear support vector regression to deconvolute the expression matrix of immune cell subtypes, enabling accurate estimation of immune cell abundance. This computational tool provides comprehensive expression data for 22 commonly observed immune cells, collectively known as LM22, encompassing immune cells of different cell types and functional states (14). In our analysis, we compared the differences in immunological characteristics between UC and control groups, as well as the distinct subtypes of UC. In addition, the association between BAMGs and immune cell were explored as well.

### WGCNA analysis

The Weighted gene co-expression network analysis (WGCNA) aimed to identify co-expressed gene modules and explore the association between gene networks and phenotypes, as well as identifying the hub genes in the network (15). The analysis was carried out using the “WGCNA” package. In the GSE92415 dataset, samples were first clustered by average-linkage hierarchical clustering based on the Euclidean distance using the “hclust” function, and outlier samples were removed when necessary. To select the soft-thresholding power (β), the “pickSoftThreshold” function was applied to evaluate powers from 1 to 20. An unsigned adjacency matrix was constructed using the chosen soft threshold (power = 15) and then transformed into an unsigned topological overlap matrix (TOM). The dissimilarity between genes was defined as 1-TOM. Hierarchical clustering of genes was performed using the average linkage method, and modules were identified using the dynamic tree-cutting algorithm with parameters set as “deepSplit= 2”, “minClusterSize= 30” and “pamRespectsDendro = FALSE”. Similar modules (eigengene correlation > 0.75) were merged using the “mergeCloseModules” function with a cut height of 0.25. For each module, the module eigengene (ME) was calculated using the “moduleEigengenes” function. The Pearson correlation analysis was performed to analyze between ME and the UC trait. Modules with a Pearson correlation coefficient |r| > 0.5 and *P* < 0.05 were selected for further analysis.

### Feature selection by machine learning algorithms

Firstly, to reduce the dimensionality of the variables, we employed Lasso regression analysis, Boruta feature selection, and Recursive Feature Elimination (RFE). The intersection of variables identified by these methods was selected as candidate genes for subsequent analysis.

Lasso regression constrains the sum of the absolute values of the coefficients of each variable to be less than a fixed value, thereby setting certain regression coefficients to zero and retaining only the most important variables (16). Additionally, Boruta analysis evaluates the importance of variables in the model and automatically filters out irrelevant features, effectively reducing the dimensionality of high-dimensional data with strong robustness, anti-overfitting, and little influence of multicollinearity (17). Furthermore, the RFE algorithm identifies the optimal subset of features by iteratively selecting and eliminating features, which enhances the simplicity and generalization ability of the model while aiding in the identification of the most relevant features (18). The intersection genes identified by these three algorithms were taken as candidate variables for machine learning analysis.

### Importance identification by machine learning algorithms

Based on the results of feature selection, eight machine learning algorithms were further employed to identify the important variables. The random forest (RF) algorithm features high accuracy, wide applicability, strong nonlinear analysis capability, and low overfitting. It predicts by using multiple independent decision trees built on randomly selected samples and attributes (19). Support vector machine (SVM) uses a hinge loss function with a regularization term to optimize structural risk, and can handle nonlinear classification via kernel methods (20). Extreme gradient boosting (XGB) is an ensemble method that combines multiple decision tree weak learners to iteratively improve prediction accuracy (21). Generalized linear models (GLM) extend linear models by applying a nonlinear link function to map linear predictions to actual responses (22). Gradient boosting machine (GBM) iteratively trains weak learners to optimize a loss function, forming a robust ensemble model. Neural network (NNET) simulates the structure of the human brain using interconnected nodes to solve complex nonlinear problems. The K-nearest neighbors (KNN) algorithm classifies new instances based on the majority class of the K closest samples in the training set (23). Decision Tree (DT) algorithm constructs a decision tree model from the given training dataset, enabling transparent and highly interpretable classification of instances (24).

All algorithms were implemented using the “caret” package (version 6.0-94), and the optimal hyperparameters were automatically selected via 5-fold cross-validation. The parameter settings for each algorithm were as follows (1): RF: The random forest algorithm was called using method = “rf” within “caret”, relying on the “randomForest” package (version 4.7-1.1), with “ntree” =500 and the optimal “mtry” set to 6 (2). SVM: Using method = “svmRadial”, the algorithm employed the radial basis kernel function as implemented in the “ksvm” function of the “kernlab” package (version 0.9-32). The cost parameter C and the kernel width sigma were automatically tuned, yielding optimal values of C = 0.5 and sigma = 0.122 (3). XGB: with method = “xgbDART”, the model was built using the “xgboost” package (version 1.7.5.1) and the “xgb.train” function, incorporating the DART (Dropouts meet Multiple Additive Regression Trees) boosting technique. Hyperparameters were automatically optimized via cross-validation, resulting in the following optimal settings: “nrounds” = 100, “max_depth” = 2, “eta” = 0.4, “subsample” = 0.5, “colsample_bytree” = 0.8, “rate_drop” = 0.01, “skip_drop” = 0.95, and “min_child_weight” = 1 (4). GLM: using method = “glm”, the “glm” function from the “stats” package was called with family = “binomial” to perform binary logistic regression (5). GBM: Using method = “gbm”, the algorithm was based on the “gbm” package (version 2.1.8.1). The number of iterations (“n.trees”), tree depth (“interaction.depth”), and learning rate (“shrinkage”) were automatically tuned, yielding optimal values of “n.trees” = 150, “interaction.depth” = 2, “shrinkage” = 0.1, and “n.minobsinnode” = 10 (6). KNN: using method = “knn”, the “knn” function from the “class” package (version 7.3-22) was called. The optimal number of neighbors k was selected by cross-validation, resulting in “k” = 9 (7). NNET: Using method = “nnet”, a single-hidden-layer feedforward neural network was constructed based on the “nnet” package (version 7.3-19). The number of hidden nodes (“size”) and weight decay (“decay”) were automatically optimized, with the optimal “size” = 3 and “decay” = 1e-04 (8). DT: Using method = “rpart”, the decision tree algorithm called the “rpart” function from the “rpart” package (version 4.1.23). The complexity parameter (“cp”) that controls tree size was automatically selected, with the optimal “cp” = 0.2.

During the process, to evaluate the importance of each gene, we applied the permutation importance method using root mean square error (RMSE) as the loss function. Because RMSE is sensitive to deviations in predicted probabilities, permuting a gene’s expression values and measuring the subsequent increase in RMSE directly quantifies the gene’s contribution to the accuracy of probabilistic predictions. Specifically, a larger RMSE increase indicates greater dependence of prediction accuracy on that gene. R package “DALEX” was utilized to uniformly calculate RMSE for each algorithm. Then, the residual distribution and feature significance were displayed. The “pROC” software package was used to draw ROC curves to evaluate the performance of the machine learning algorithms.

### Unsupervised cluster and GSVA analysis

Based on the feature genes identified by above machine learning algorithms, the unsupervised cluster analysis was conducted by the “ConsensusClusterPlus” package. The ConsensusClusterPlus algorithm calculates the most stable clustering result by conducting multiple random sampling and clustering operations on the dataset (25, 26). In our analysis, the parameter “maxK” was selected 9, “pItem” was selected 0.8, “pFeature” was set 1, “clusterAlg” was selected “km,” and the “distance” was selected “Euclidean”. The optimal cluster number was determined based on the combination of cumulative distribution function (CDF) curve, consistency matrix and consistency cluster score>0.8. Subsequently, the principal component analysis (PCA) was performed after clustering. The PCA analysis could evaluate the effect of clustering by mapping the clustering results into a low-dimensional space and observing the distribution between different categories (27).

To further explore the biological function of distinct clusters, we performed the GSVA analysis by using the “gsva” package. Enrichment of molecular pathways across different samples were evaluated by converting gene expression into gene set expression matrix (28). In our analysis, the “c2.cp. kegg. v7.5.1. symbols. gmt” and “c5.go.v7.5.1.symbols.gmt” gene sets were selected as the reference gene sets.

### Single cell analysis

To further investigate the significance of feature genes at the single-cell level, we conducted single cell analysis on the GSE214695 dataset, which included 6 healthy control and 6 UC samples. First, data were evaluated using the “Seurat” package, and low-quality cells were filtered with parameters set to min.cells = 3, min.features = 200, the content of mitochondrial RNA in each cell was required to be less than 15% and HB_percent <5%. Then, doublets were identified and removed using the “scDblFinder” package. Data were normalized by using the “NormalizeData” function. Principal component analysis (PCA) was applied on the top 3000 highly variable genes (HVGs) and batch effects during dimensionality reduction were mitigated using the “Harmony” method. Subsequently, nonlinear dimensional reduction on high-dimensional data was performed using the tSNE algorithm. Cluster markers were identified using “FindAllMarkers” function, and major cell types were manually annotated based on canonical marker genes and literature. Furthermore, the “AddModuleScore” function from the “Seurat” package was used to calculate the average expression scores for the identified BAMGs gene sets, and the Wilcoxon test was applied to compare differences across different cell types. Finally, cell-cell communication analysis was performed using the “CellChat” package. Briefly, a CellChat object was created with normalized expression data and cell type labels. Based on the human CellChatDB, overexpressed ligand-receptor pairs were identified, and communication probabilities were computed using the default algorithm, with low-confidence interactions filtered (min.cells = 10). Ligand-receptor pairs were then aggregated into signaling pathways, and the overall communication network was built and visualized.

### Establishment model of DSS-induced colitis in mice

In our study, 6–8week-old female C57BL/6 J mice (18–22g) were purchased from GemPharmatech Co., Ltd, Nanjing, China. Before the experiment, mice were housed in the SPF animal laboratory in Nanjing Medical University and underwent a one-week acclimation period. Subsequently, the mice were randomly divided into two groups: control group and the DSS group. The dextran sodium sulfate was purchased from MedChemExpress, Nanjing, China, lot: HY-116282. Mice in the DSS group were administered a 3% DSS solution orally for 7 days, while those in the control group received normal drinking water. During the experiment, the body weight of the mice was recorded daily, and fecal samples were collected for occult blood testing, and changes in the Disease Activity Index (DAI) scores of both groups were monitored. On day 8 of the experiment, the mice were sacrificed, and colon tissues, spleens were obtained and stored at −80 °C for subsequent analysis. Mice were humanely euthanized via slow carbon dioxide (CO_2_) inhalation at a flow rate of 30% of the chamber volume per minute, following the local standard animal ethical protocols. The animals were confirmed to be unconscious and devoid of vital signs before tissue collection and further experimental manipulation. All animal experiments were conducted in accordance with the procedures approved by the Ethical Review Committee for Laboratory Animal Welfare of Nanjing Medical University (IACUC-2012011).

### Western blot analysis

The total protein was extracted from the tissue samples after lysis and fragmentation. Protein concentration was determined, and samples were denatured by boiling in SDS loading buffer. Proteins were separated via SDS-PAGE gel electrophoresis and subsequently transferred onto PVDF membranes. Membranes were blocked with 5% skim milk for 1 hour at room temperature, followed by incubation with specific primary antibodies overnight at 4°C and secondary antibodies for 1 hour at room temperature. Between each step, the membranes were washed three times 10min with TBST. primary antibodies were purchased from Co. Abmart. Mouse SLC23A1 antibody (lot:PH7038, WB 1:1000), PHYH antibody (PS18209, WB 1:5000) and CH25H antibody (PS11386, WB 1:1000). ECL chemiluminescence substrates were used for signal development, and protein expression levels were quantified by normalizing gray values to GAPDH as internal references.

### Immunohistochemical (IHC) analysis

The colon tissues from each group of mice were collected, fixed in 4% formaldehyde, and embedded in paraffin. Antigen retrieval was performed using sodium citrate buffer at 80 °C for 15–20 minutes, followed by PBS washing. Endogenous peroxidase activity was blocked with 3% H_2_O_2_, and sections were incubated with primary antibodies overnight at 4°C after blocking with 5% goat serum. After secondary antibody incubation and further PBS washes, DAB staining was conducted. Images were analyzed using Motic EasyScan slide scanner (Xiamen Moody Medical Diagnostic System Co., LTD.).

### RT-qPCR analysis for peripheral blood mononuclear cells (PBMCs)

Total RNA was extracted from 32 clinical UC patients’ peripheral PBMCs and 30 healthy controls using the Trizol reagent (Thermo Fisher Scientific, Germany). Reverse transcription and qPCR were performed according to the method recommended by the kit manufacturer (Vazyme Biotechnology Co., Nanjing, China). The SYBR Green PCR kit (Vazyme, cat: Q121-02) was utilized and the analysis was conducted using the ABI Applied Biosystems 7500 PCR quantitative instrument (Thermo Fisher Scientific, Germany). Samples were analyzed in duplicate across all groups and β- actin was the reference for the relative quantitative. The primers were shown in **Supplementary Table 3**. The relative mRNA expression levels were quantified using the 2-ΔΔCq method. This study was approved by the Ethics Committee of the First Affiliated Hospital of Nanjing Medical University (2025-SR-1243) and in accordance with the principles of the Declaration of Helsinki.

## Results

### Identification of DEGs

**Figure 2:**
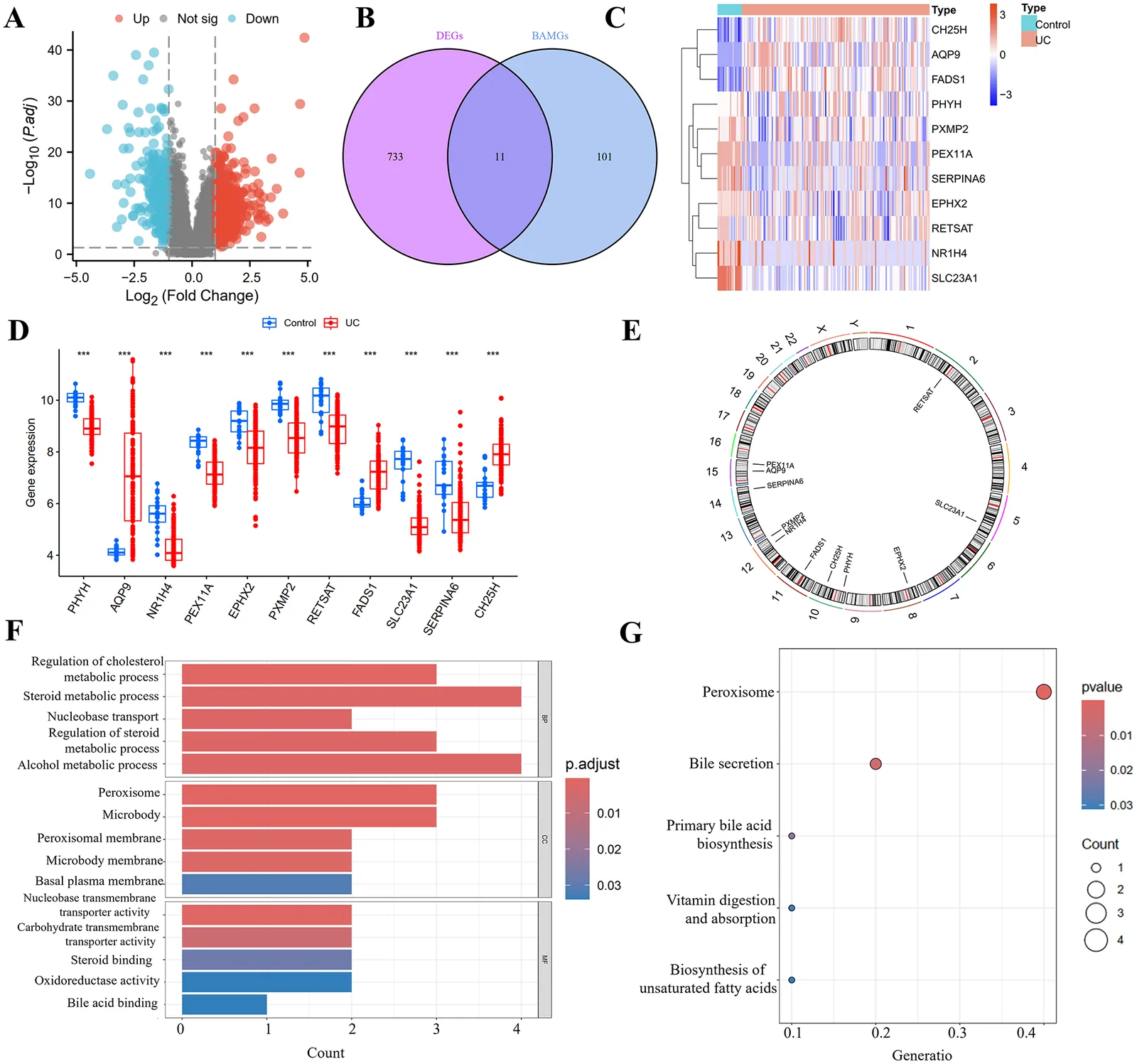
Identification of differential BAMGs in UC. **(A)** The volcano plot showed differentially expressed genes in UC, with red dots representing up-regulated genes and blue dots representing the down-regulated genes. **(B)** Venn diagram suggested that 11 BAMGs overlapped with differentially expressed genes. **(C)** Heatmap of expression of 11 BAMGs in UC and controls. **(D)** Boxplot of expression of 11 BAMGs in UC and controls. **(E)** Location of the 11 BAMGs on the chromosome. **(F)** GO enrichment analysis of 11 BAMGs. **(G)** KEGG pathway enrichment analysis of the BAMGs.

Based on the criteria of |log2FC| > 1 and adjust *P* < 0.05, a total of 744 DEGs were identified, including 473 up-regulated and 271 down-regulated genes (**Figure 2A**). In addition, a total of 11 BAMGs had intersection with the DEGs (**Figure 2B**), including *PHYH*, *AQP9*, *NR1H4*, *PEX11A*, *EPHX2*, *PXMP2*, *RETSAT*, *FADS1*, *SLC23A1*, *SERPINA6* and *CH25H* (**Figure 2C**). Of which, 3 genes (*AQP9*, *FADS1*, *CH25H*) were significantly up-regulated in UC samples compared with the controls, while 8 genes (*PHYH*, *NR1H4*, *PEX11A*, *EPHX2*, *PXMP2*, *RETSAT*, *SLC23A1*, *SERPINA6*) were significantly down-regulated (**Figure 2D**). The chromosomal locations of these genes were depicted in **Figure 2E**.

### Functional enrichment analysis

To further elucidate the biological functions of these differentially expressed BAMGs, GO and KEGG enrichment analyses were conducted. The results indicated that these 11 BAMGs were associated with biological processes (BP) including regulation of cholesterol metabolic process, steroid metabolic process, and nucleobase transport (**Figure 2F**); cellular components (CC), such as peroxisome and microbody (**Figure 2F**); and molecular functions (MF), such as nucleobase transmembrane transporter activity, steroid binding, and oxidoreductase activity (**Figure 2F**). Moreover, KEGG pathway enrichment analysis revealed that these BAMGs were implicated in signaling pathways including peroxisome, bile secretion, and primary bile acid biosynthesis (**Figure 2G**).

### Immune cell infiltration analysis

To elucidate the differences in immunological characteristics between UC and control groups, the CIBERSORT algorithm was used to show differences in the proportions of 22 infiltrating immune cell types in the GSE92415 dataset. The results were shown in **Supplementary Material**, **Supplementary Figure 1**. The UC group had a higher proportion of activated CD4 T cells, gamma delta T cells, M0 macrophages, M1macrophages, activated dendritic cells, activated mast cells and neutrophils than the control group (*P* < 0.001), while the proportions regulatory T cells, M2 macrophages, and resting mast cells were lower (*P* < 0.001).

Furthermore, the correlations between the expression of the 11 BAMGs and the abundance of immune cells were explored by spearman analysis. The results revealed a complex regulatory relationship (**Supplementary Figure 1C**). For instance, the expression of *SLC23A1* showed the negative association with M1 macrophages (*P* < 0.001) and neutrophils (*P* < 0.001), but positive regulatory relationship with M2 macrophages (*P* < 0.001) and monocytes (*P* < 0.001). These findings suggested a potential interplay between BAMGs and immune cells that may contribute to UC pathogenesis.

### WGCNA analysis

**Figure 3:**
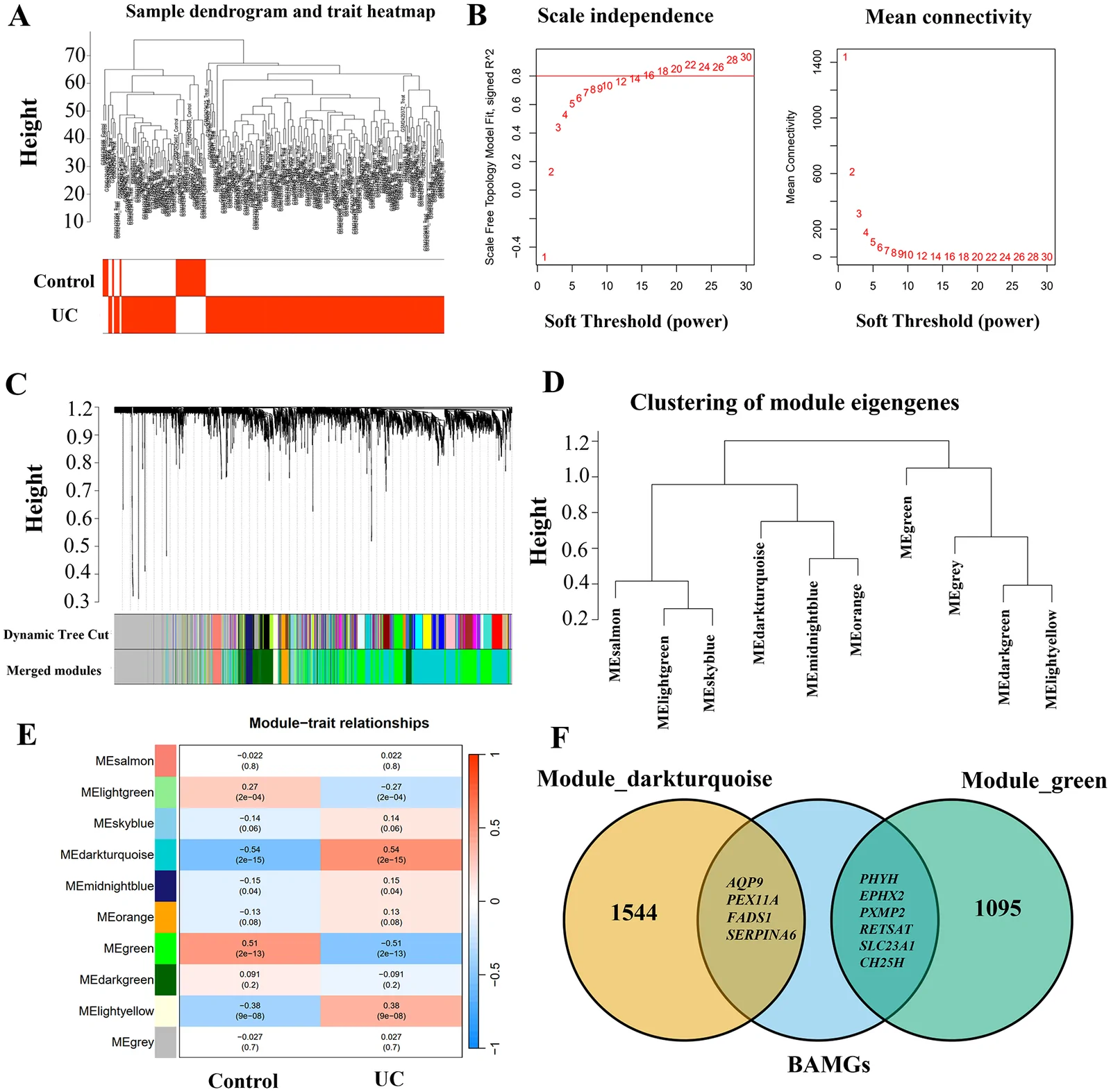
Results of WGCNA analysis. **(A)** The sample dendrogram and feature heatmap were generated based on the Euclidean distance by using the average clustering method for hierarchical clustering of samples. **(B)** Selection of the best soft threshold. **(C)** Gene hierarchy tree clustering diagram. **(D)** Ten gene modules were obtained through the dynamic cutting method. **(E)** Heatmap showed the relations between the module and features. **(F)** The Venn diagram of the intersection of the BAMGs and WGCNA analysis modules.

To identify key BAMGs associated with UC, WGCNA was performed on the GSE91425 dataset. Genes within the top 25% of expression variance (4,974 genes) were retained for analysis. First, samples were clustered to detect outliers, and a height cut-off of 80 was applied to exclude obvious aberrant samples (**Figure 3A**). A soft-thresholding power of β = 15 was selected to construct an unsigned network and topological overlap matrix (**Figure 3B**). Using the dynamic tree-cutting method with a minimum module size of 30 and merging similar modules at a height cut of 0.25, ten co-expression modules were identified (**Figures 3C, D**). Correlation analysis revealed that the darkturquoise module (1,548 genes) was significantly positively correlated with UC (*r* = 0.544, *P* = 2.16e-15), while the green module (1,101 genes) showed a significant negative correlation (*r* = –0.512, P = 1.50e-13) (**Figure 3E**). Analysis by Venn diagram indicated that the darkturquoise module shared 4 intersection genes with differential BAMGs, and the green module shared 6 intersection genes (**Figure 3F**). Given that both the darkturquoise module (positive correlation) and the green module (negative correlation) showed significant associations with UC, the intersecting genes between these two modules and differential BAMGs were combined in our analysis, resulting in differential unique genes for subsequent analysis.

### Analysis of machine learning algorithms

**Figure 4:**
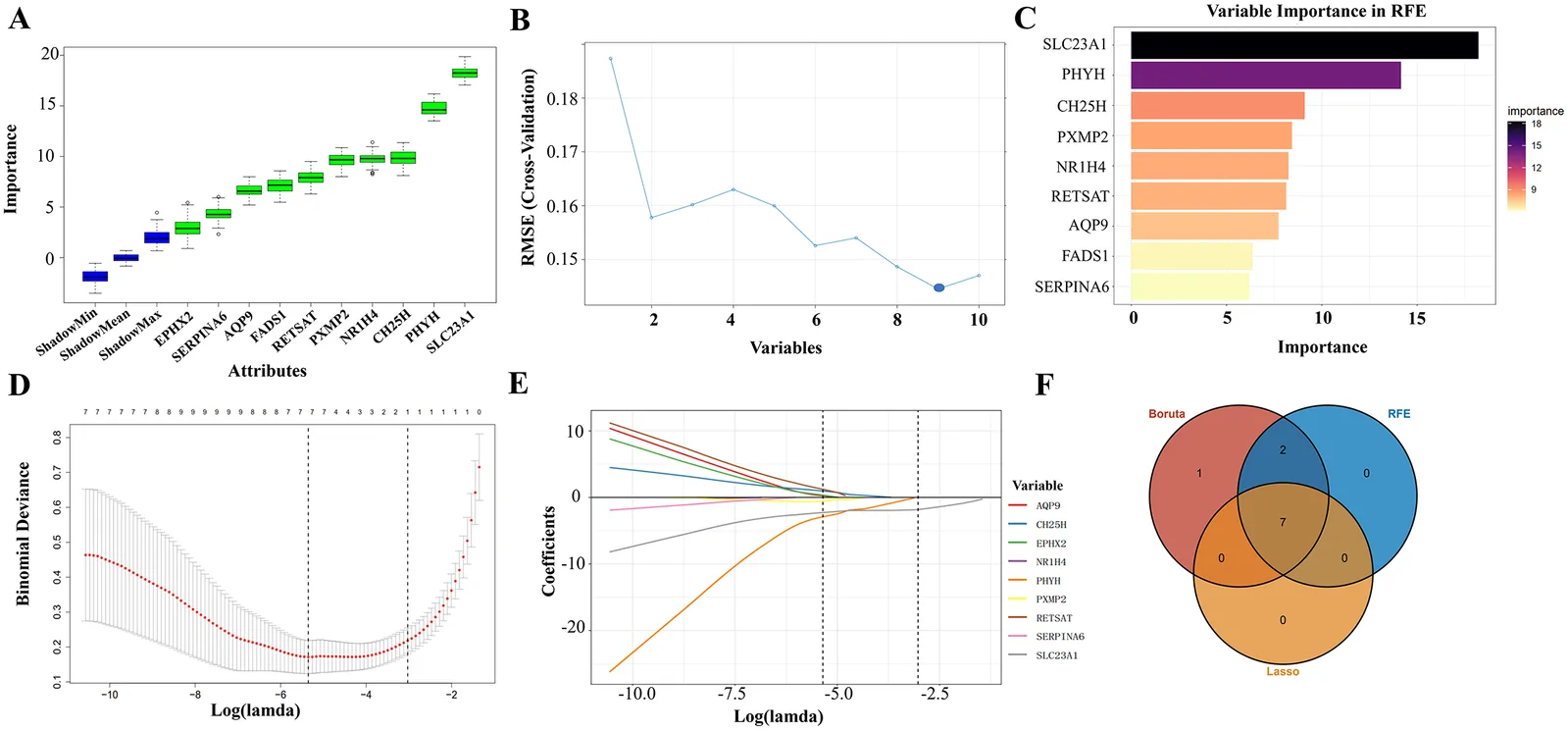
Feature selection Based on machine learning algorithms. **(A)** Boruta analysis. **(C)** RFE analysis. **(C)** Distribution plots of important variables screened by RFE analysis. **(D)** Lasso regression analysis. **(D)** Cross validation curve by lasso regression analysis. **(F)** The Venn diagram of the intersection of the BAMGs through the three machine learning algorithms.

Based on the WGCNA results, the ten BAMGs identified above were subjected to rigorous feature selection using three algorithms. First, the Boruta analysis revealed the importance distribution of these 10 genes. In this algorithm, none of the variables were rejected, and all 10 genes were confirmed as relevant, as illustrated in **Figure 4A**. Second, recursive feature elimination (RFE) algorithms identified nine genes as optimal for model performance, as evidenced by the minimal root mean square error (RMSE) at this dimensionality (**Figure 4B**); the corresponding variable importance ranking was shown in **Figure 4C**. Third, Lasso regression yielded a seven-gene signature—comprising *PHYH*, *AQP9*, *EPHX2*, *PXMP2*, *RETSAT*, *SLC23A1* and *CH25H*—that minimized prediction error (**Figures 4D, E**). Therefore, based on the feature selection algorithms, we selected the seven intersection genes (*PHYH*, *AQP9*, *EPHX2*, *PXMP2*, *RETSAT*, *SLC23A1* and *CH25H*) for subsequent analysis (**Figure 4F**).

**Figure 5:**
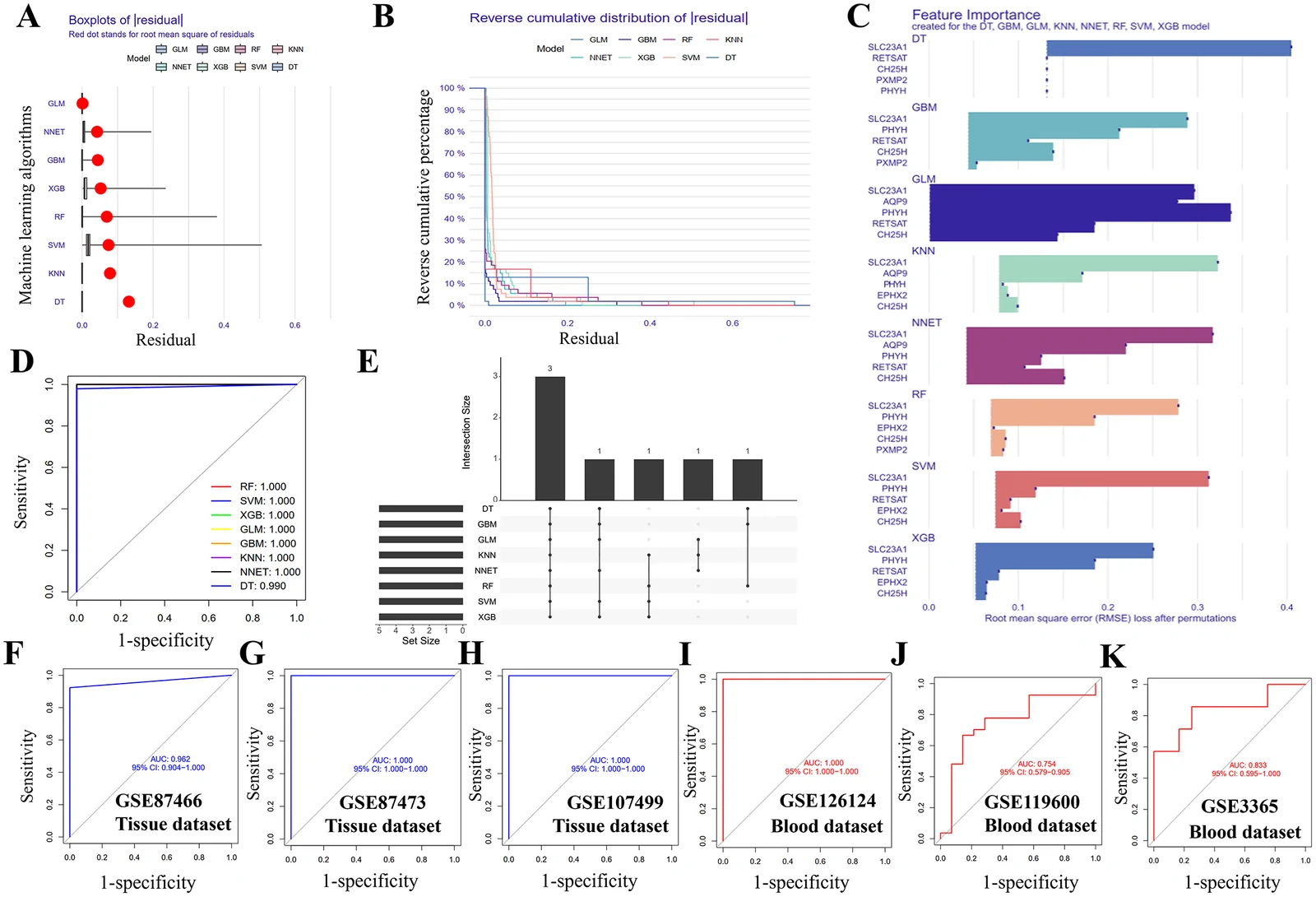
Identification of feature BAMGs. **(A)** Box plot showed the residuals for each machine learning algorithms, the red dot represents the root mean square of the residual. **(B)** Cumulative residual distribution of each machine learning algorithms. **(C)** Important features in each machine learning algorithms. **(D)** ROC analysis. **(E)** Upset plot showed that three feature genes overlapped in all machine learning algorithms. Validation analysis of external datasets **(F)** GSE87466, **(G)** GSE87473, **(H)** GSE107499, **(I)** GSE126124, **(J)** GSE119600, **(K)** GSE3365.

Subsequently, 8 machine learning algorithms (GLM, NNET, GBM, XGB, RF, SVM, KNN and DT) were applied to prioritize feature variables. Cumulative residual distributions for all models were shown in **Figures 5A, B**. To identify variable importance, the top five features ranked by permutation-based importance were extracted from each algorithm (**Figure 5C**). Model performance was rigorously evaluated using five-fold cross-validation, with ROC curves and corresponding area under the curve (AUC) values presented in **Figure 5D**. All eight models achieved exceptional discriminative accuracy, confirming robust predictive capacity across diverse algorithmic frameworks. In order to identify the most important variables, we computed the intersection of the top five most important variables from each model, which yielded three consistently prioritized genes: *CH25H*, *SLC23A1* and *PHYH* (**Figures 5C, E**). External validation revealed robust diagnostic capabilities of the three genes—in the UC tissue data sets GSE87466, GSE87473 and GSE107499, ROC curve analysis showed that the AUC of the three genes was 0.962,1,1, respectively (**Figures 5F–H**). In addition, we also explored three peripheral blood datasets in UC patient GSE126124, GSE119600 and GSE3365, with ROC curve areas of 1, 0.754 and 0.883, respectively (**Figures 5I–K**).

### GSEA and Immune analysis of feature genes

**Figure 6:**
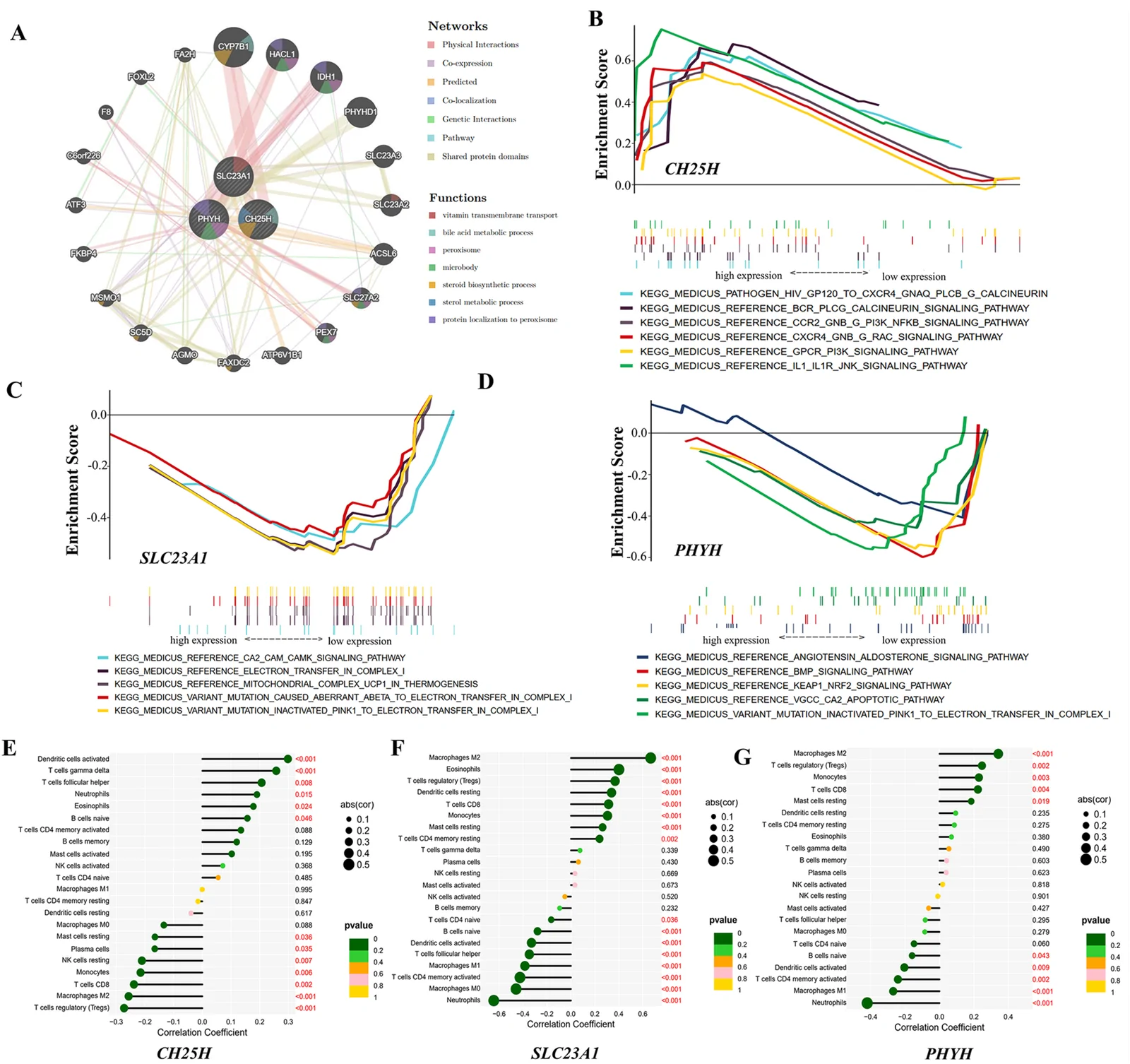
GSEA and immune cell analysis for feature genes. **(A)** Protein interaction network analysis. GSEA analysis for **(B)** *CH25H*, **(C)** *SLC23A1*, **(D)** *PHYH.* Correlation analysis between feature genes and immune cell abundance **(E)** *CH25H*, **(F)** *SLC23A1*, **(G)** *PHYH*.

In this study, we employed GeneMANIA database to investigate protein–protein interaction (PPI) and co-expression networks centered on the three BAMGs. As shown in **Figure 6**, a total of 20 high-confidence interacting partners were identified (**Figure 6A**). In addition, GSEA analysis showed that high expression of *CH25H* was enriched in the CXCR4, PI3K, NF-κB and IL1_IL1R_JNK pathway, etc (**Figure 6B**). In *SLC23A1* low-expression subgroup, pathways such as CA2_CAM_CAMK signaling pathway, electron transfer in complex I and mitochondrial complex UCP1 in thermogenesis was enriched in etc. (**Figure 6C**); moreover, low expression of *PHYH* was enriched in angiotensin aldosterone signaling pathway, BMP signaling pathway and KEAP1_NRF2 signaling pathway, etc. (**Figure 6D**). Collectively, these findings implicated the three genes in key inflammatory, metabolic, and redox regulatory processes.

To further elucidate immunological context, we assessed correlations between the BAMGs expression and immune cell abundances using CIBERSORT. Results showed that the *CH25H* gene was positively correlated with activated dendritic cells, neutrophils and naive B cells, etc. However, it was negatively correlated with regulatory T cells, CD8 T cells, M2 macrophages, etc. (**Figure 6E**). In contrast, *SLC23A1* expression was positively correlated with M2 macrophages, eosinophils, regulatory T cells, CD8 T cells, monocytes, but negatively correlated with neutrophils, M0/M1 macrophages, etc. (**Figure 6F**). Similarly, *PHYH* expression showed positive association were found with the M2 macrophages, regulatory T cells as well as CD8 T cells, while negative association were observed with neutrophils, M0/M1 macrophages, activated memory CD4 T cells, naive B cells, etc. (**Figure 6G**). The results suggested that the association between key genes and immune cells may play the important role in the development of UC.

### Single cell analysis

**Figure 7:**
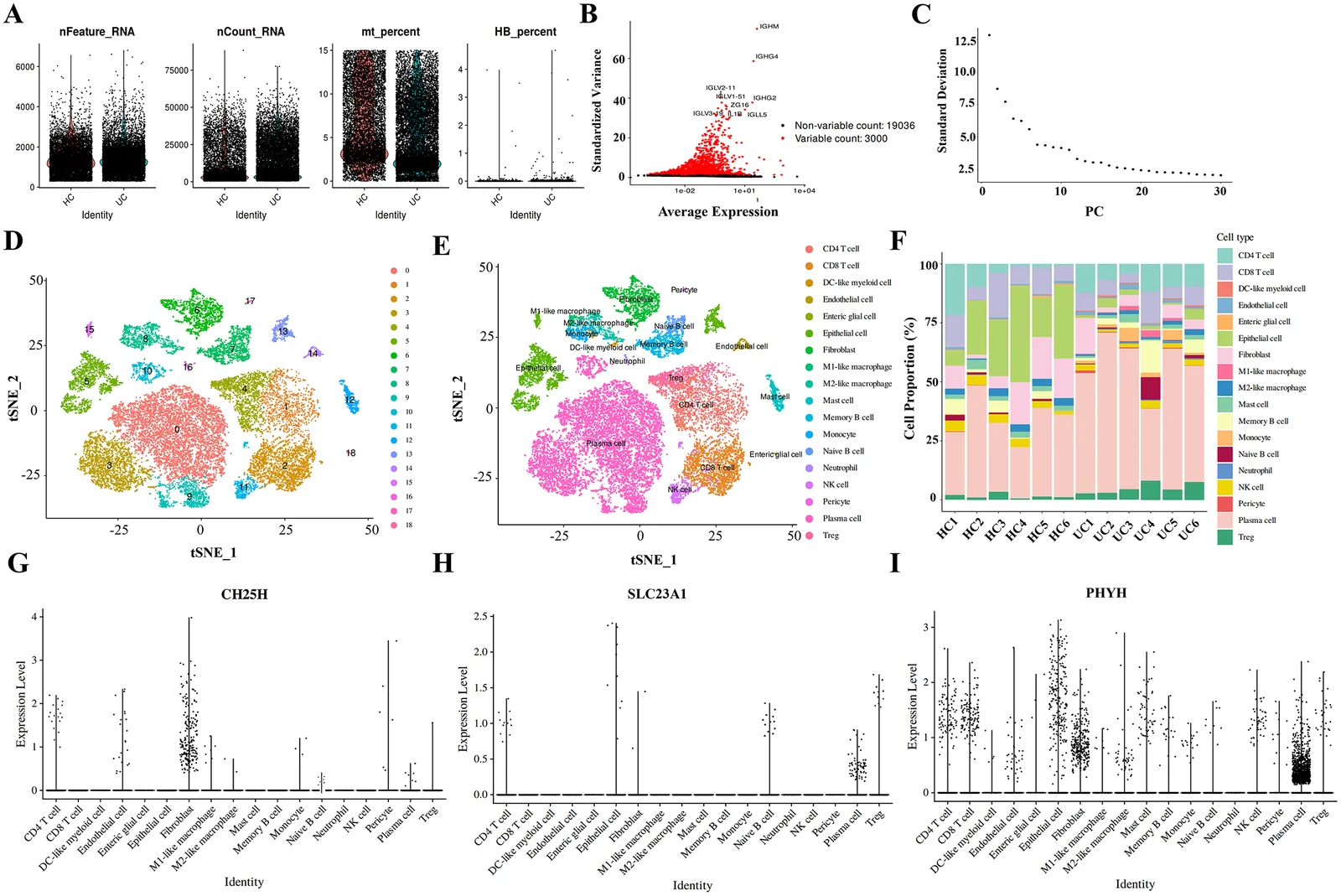
Expression of feature genes at single cell analysis. **(A)** Results of cell quality control. **(B)** Identification of 3000 genes exhibiting high variability. **(C)** Elbow plot. **(D)** t-SNE map showed the distribution of 18 type of cell subpopulations. **(E)** t-SNE plot of cell annotation results. **(F)** The expression abundance of each cell type in samples; **(G)** Expression of *SLC23A1* at single cell; **(H)** Expression of *CH25H* at single cell; **(I)** Expression of *PHYH* at single cell.

**Figure 8:**
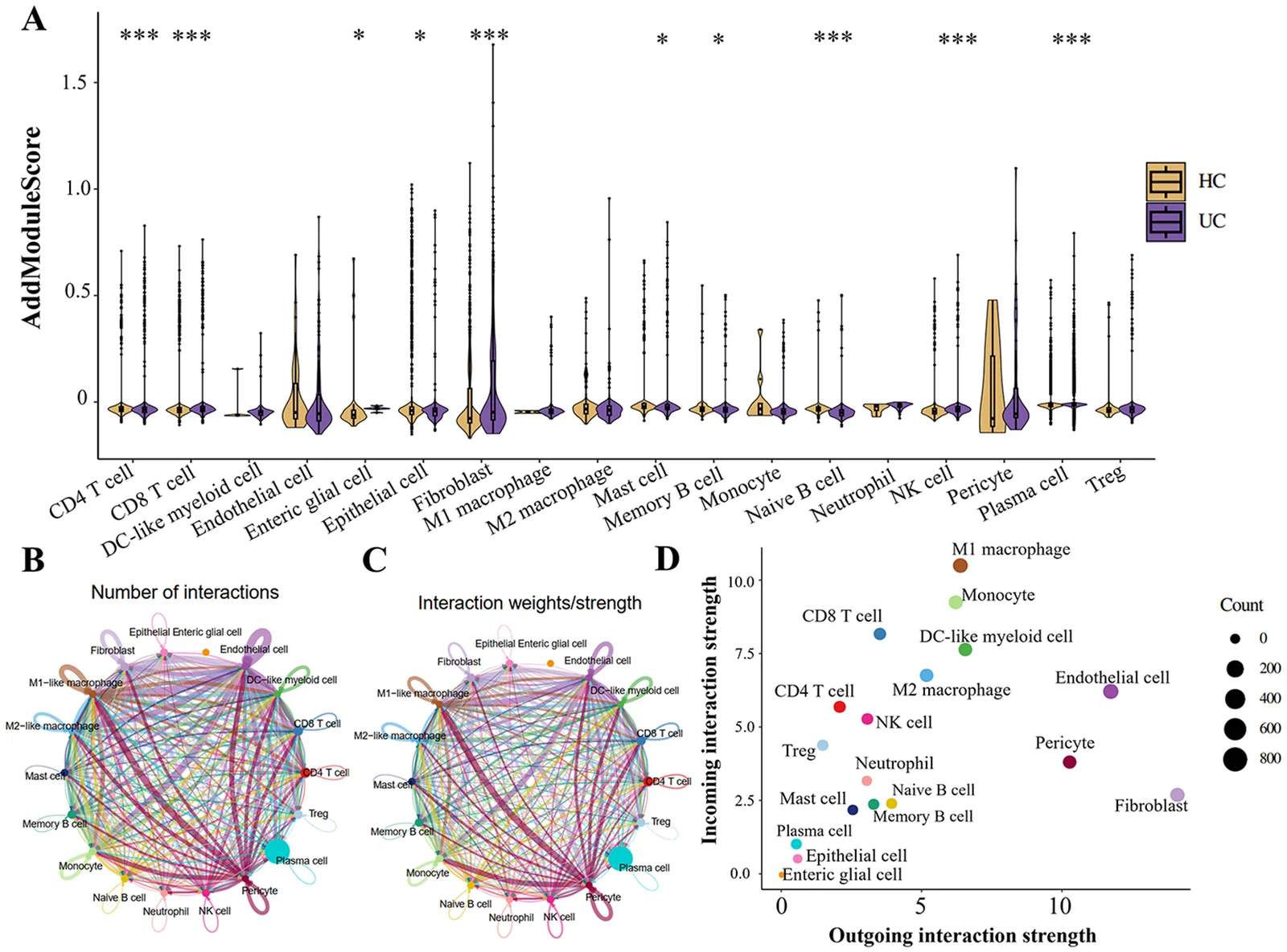
Results of cell scoring and cell communication. **(A)** AddModuleScore function to calculate the scores of feature genes for each cell type. *P<0.05, ***P<0.001. **(B)** Cell communication analysis showed the number of interactions. **(C)** Cell communication analysis showed interaction weights/strength. **(D)** Signaling role plot showed the intensity of signal emission and reception for each cell type.

We performed single-cell analysis of six UC samples and healthy controls from the GSE214695 dataset, and the results were presented in **Figure 6**. After analysis of quality control and data filtering (**Figure 7A**), 3000 genes exhibiting high variability were identified (**Figure 7B**). Principal components were identified by PCA analysis (**Figure 7C**), followed by nonlinear dimensionality reduction based on the first 30 dimensions of the Harmony-reduced results. Cell types were manually annotated based on canonical marker genes and the literature (**Supplementary Material**, **Supplementary Figure 2**). Cluster analysis identified a total of 18 distinct cell subsets (**Figures 7D, E**). Cell-type proportion analysis revealed that B-lineage cells (including naïve B cells and plasma cells) and T-lineage cells (including CD4 T cells, CD8 T cells, and Tregs) constituted predominant immune populations in UC samples (**Figure 7F**). Analysis of the expression of biomarkers in different cell types showed that *CH25H* were differentially expressed CD4 T cell, naïve B and plasma cells (**Figure 7G**); *SLC23A1* were differentially expressed CD4 T cell, naïve B, plasma cells and Tregs (**Figure 7H**); whereas the *PHYH* showed broad expression, especially in plasma cells, CD4 T and CD8 T cells (**Figure 7I**). In addition, we utilized the AddModuleScore function to calculate the scores of the identified BMAGs for each cell type. Results revealed significant differences in BMAGs scores in CD4 and CD8 T cells, as well as in memory B cells, naïve B cells, and plasma cells (**Figure 8A**). Collectively, these findings suggested the feature genes may contribute to UC pathogenesis primarily through modulation of T- and B-cell functions. Furthermore, the analysis of intercellular communication revealed the complex interactions occurring in the UC environment (**Figures 8B–D**).

### Unsupervised clustering of UC patients

Based on the three feature BAMGs,162 UC patients in the GSE92415 were divided into distinct clusters (**Supplementary Figure 3**). According to the CDF curve, the relative change in the area of the CDF curve, as well as the consistent clustering score, we selected k = 2 as the best value for further analysis (**Supplementary Figures 3A–C**). Therefore, 162 UC patients were divided into two different subtypes, with 91 samples in cluster 1 (C1) and 71 samples in cluster 2 (C2). The expression patterns of the feature BAMGs of the two clusters were shown in **Supplementary Figure 3D**. Immune cell infiltration analysis showed that C1 exhibited a higher proportion of M1 macrophages, neutrophils, activated DC cells, while C2 samples had a higher proportion of CD8 T cells, monocyte, M2 macrophages, etc. (**Supplementary Figure 3F**).

Moreover, GSVA analysis revealed that C2 exhibited enhanced enrichment in biological processes such as positive regulation of vascular endothelial growth factor production, regulation of cell proliferation, and positive regulation of lymphocyte migration, as well as pathways including the chemokine signaling pathway, cytokine-cytokine receptor interaction, and intestinal immune network for IgA production (**Supplementary Figures 3G, H**). Overall, C2 may represented a chronic, adaptive immune-dominant subtype, characterized by persistent and chronic immune activation, accompanied by mucosal immune disorders and tissue remodeling. Hence, there existed discernible disparities in immune status and molecular pathways between the two subtypes of UC, which could hold substantial implications for tailored clinical diagnosis and treatment.

### Altered expression of feature gene in DSS-induced colitis

**Figure 9:**
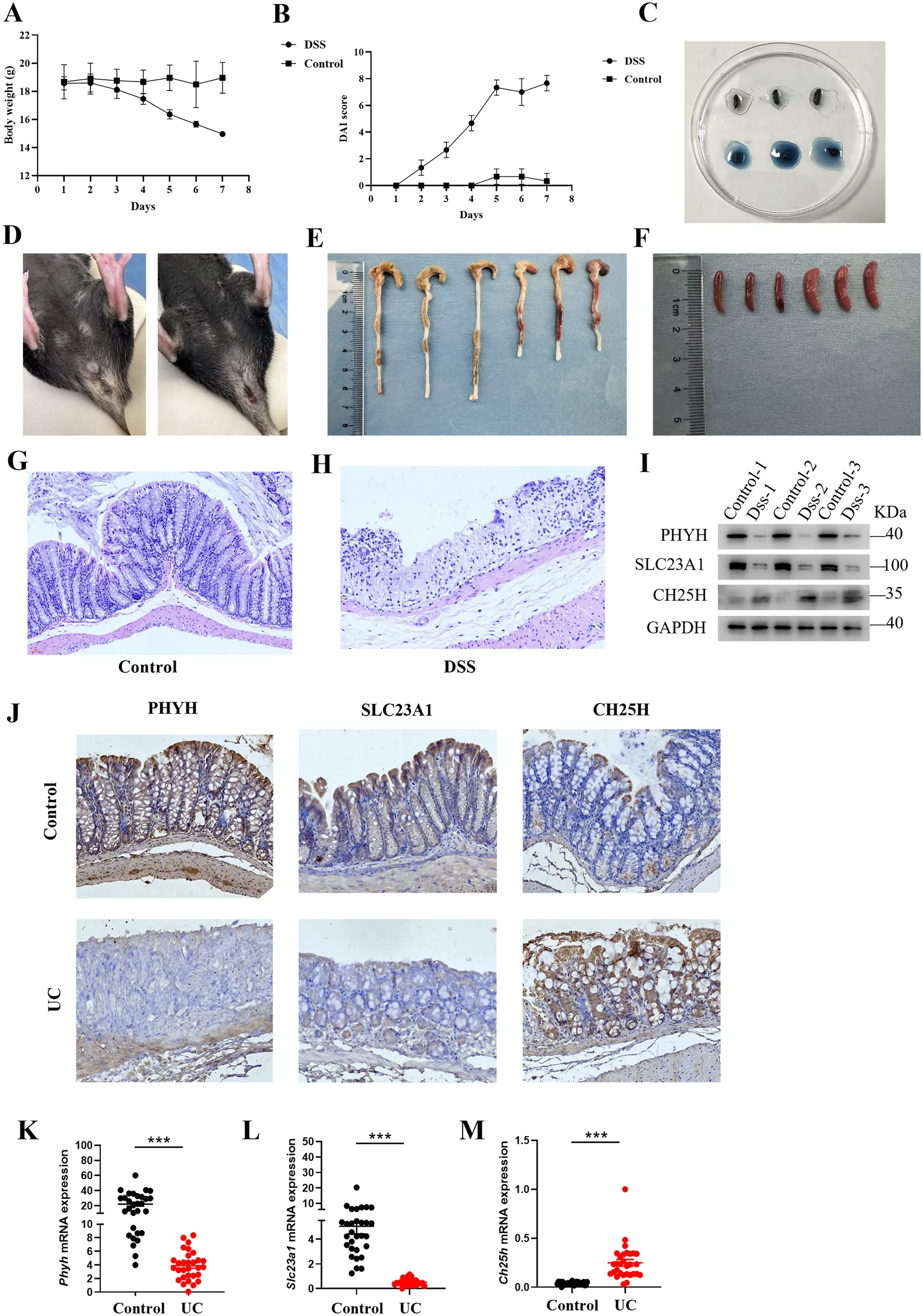
Results of DSS-induced colitis model in mice. **(A)** Daily changes in body weight of mice. **(B)** Daily changes in DAI scores. **(C)** Results of fecal occult blood test. **(D)** Representative images of bloody diarrhoea, mice on the left were the control group and the mice on the right were the DSS group. **(E)** Colon tissues of control and DSS mice, results showed that the colon length of mice in DSS group was significantly shortened. **(F)** Spleen tissues of control and DSS group, result showed that the spleen of DSS group was significantly larger. **(G, H)** Results of histological sections stained with hematoxylin and eosin (HE). **(I)** Western blot analysis for detection of protein expression. **(J)** Immunohistochemical analysis revealed the expression of *CH25H*, *SLC23A1* and *PHYH* in the colonic tissues of UC mice. Results of RT-qPCR analysis in clinical UC patients’ blood samples. RT-qPCR analysis of **(K)** *PHYH*; **(L)** *SLC23A1*; **(M)** *CH25H* in clinical UC blood samples (PBMCs) and controls. ***P<0.001.

During DSS-induced colitis in mice, the body weight changes in control and DSS groups were presented in **Figure 9A**. The DAI scores of DSS group mice increased significantly during the course of the experiment (**Figure 9B**). **Figure 9C** showed the fecal occult blood test results of the two groups of mice on the fourth day of the experiment, and the feces of mice in the DSS group all showed macroscopic blue color. Compared with the control group, a typical macroscopic image of mice in the DSS group showing bloody intestines were shown in **Figure 9D**. After the ninth day of the experiment, mice were sacrificed, and the colon of both groups of mice was dissected as shown in **Figure 9E**. Compared with the control group, the mice in the DSS group had lower colon length. In addition, the spleens of mice in the DSS group showed larger volumes (**Figure 9F**).

Histological analysis showed that the colon of mice with colitis showed loss of crypt glands, mucosal damage and inflammatory cell infiltration, while the control maintained intact colonic mucosa and clear crypt structure (**Figures 9G, H**). The results indicated the acute experimental colitis model was successfully established. Western blot analysis showed that the protein expression levels of SLC23A1 and PHYH were decreased, while the CH25H protein was increased in the DSS group (**Figure 9I**). In addition, immunohistochemical analysis also revealed consistent findings (**Figure 9J**).

### RT-qPCR analysis for clinical UC patients

To further validate the expression of key genes in UC, we performed RT-qPCR analysis on 32 clinical UC patients and 30 healthy controls. The basic clinical characteristics of the patients and healthy controls were shown in **Supplementary Table 4**. Compared with the control group, the levels of WBC (*P* = 0.018), neutrophils (*P* = 0.013), platelets (*P* = 0.012), and ESR (*P* < 0.001) were significantly higher in the UC group, while the levels of TP (*P* < 0.001) and ALB (*P* < 0.001) were significantly lower. Result of the RT-qPCR analysis showed that the expression level of *CH25H* gene was significantly higher in UC blood samples, while the expression levels of *SLC23A1* and *PHYH* genes were significantly lower (**Figures 9K–M**).

## Discussion

Recent evidence has demonstrated the pivotal roles of bile acids in maintaining intestinal homeostasis and regulating inflammation (29, 30). Ecological imbalances (e.g., gut dysbiosis) in IBD patients can disrupt bile acid transformation, leading to altered bile acid levels. Conversely, bile acids also modulate microbial communities, contributing to intestinal dysbiosis and impairing barrier integrity and immune function (29). Given these bidirectional interactions, bile acids are recognized as potent signaling molecules (31). In addition, the expression and function of key bile acid-activated receptors including *FXR, GPBAR1, PXR, VDR*, and *RORγt* are regulated by intestinal inflammation (32), their dysregulation promotes the polarization of intestinal T cells and macrophages toward a proinflammatory phenotype (33–35). Therefore, bile acids are involved in maintaining the integrity of the intestinal barrier, regulating gene expression, maintaining metabolic homeostasis (36).

By comprehensive bioinformatics and machine learning strategies, we successfully identified three feature genes (*CH25H*, *SLC23A1* and *PHYH*) from bile acid metabolic signaling pathway. These findings were well validated in samples from both clinical UC patients and mice models. Expression of *CH25H* was elevated in UC samples, while the *SLC23A1* and *PHYH* were decreased. Under physiological conditions, *CH25H* catalyzes the synthesis of 25-hydroxycholesterol (25-HC) to maintain cholesterol homeostasis (37). *SLC23A1*, belongs to solute carrier family 23 member 1, encoding sodium-dependent vitamin C transporter 1 (SVCT1), which is mainly involved in the absorption and reabsorption of vitamin C (38). Its expression was upregulated under bile acid accumulation and could be induced by bile acids such as chenodeoxycholic acid, suggesting a protective role (39, 40). Regarding *PHYH*, which encodes the enzyme phytanoyl-CoA hydroxylase, is critical in the formation of peroxisomal protein (41). Clinically, patients with Refsum disease caused by *PHYH* deficiency often present with bile acid metabolism disorders (42).

In our analysis, unsupervised clustering based on the expression profiles of the three BAMGs stratified UC samples into two biologically distinct subgroups: cluster 1 exhibited a higher proportion of M1 macrophages, neutrophils, activated DC cells, while cluster 2 showed a higher proportion of M2 macrophages, CD8 T cells. Consequently, C1 may manifest as a rapid destructive inflammation dominated by neutrophils and M1 macrophages, while C2 may represent a chronic, adaptive immune-dominated subtype of UC. These results indicated that the three-feature BAMGs signature served not only as potential biomarkers for UC but also as a potential molecular subtyping framework to guide personalized decisions.

Studies of *CH25H* have revealed its dual role in intestinal inflammation. Yang et al. (43) reported a strong correlation between colonic 25-HC levels in DSS mice and NLRP3 inflammasome components, which is a critical node in UC pathogenesis (44). Its activation promotes the cleavage of caspase-1 and facilitates the release of inflammatory mediators such as IL-1β, thereby amplifying the intestinal inflammatory response (44). However, other studies have also identified an anti-inflammatory effect (45, 46). Sheng et al. (47) have revealed a protective role for 25-HC in DSS-induced colitis, supplementing with 25-HC can alleviate intestinal damage, increase the expression of tight junction proteins, and reduce the levels of local and systemic pro-inflammatory factors IL-6 (47). The researchers pointed out that this opposite effect of *CH25H* might be related to the microbiota conditions. In this study, GSEA analysis revealed that high expression of *CH25H* were significantly enriched in classic pro-inflammatory signaling pathways such as CXCR4, CCR2, PI3K, NF-κB and IL-1/JNK. These enriched pathways suggested that *CH25H* may be closely related to the inflammatory amplification loop of UC. Moreover, the immune infiltration analysis found that the expression of *CH25H* was significantly negatively correlated with M2 macrophages, while positively correlated with naïve B cells, neutrophils, and activated dendritic cells. At the single-cell level, we observed that *CH25H* was differentially expressed in naïve B cells, CD4 T cell and plasma cells. In contrast to the protective role reported by Sheng et al. (47), our results seemed to point a pro-inflammatory tendency of *CH25H* in UC. This discrepancy may reflect differences in disease stage, species, or microbiota conditions as suggested by Sheng et al. (47). Based on the above results, we infer that in UC, the high expression of *CH25H* may participate in the pro-inflammatory pathways, while regulating the immune inflammatory cells, thereby promoting inflammation.

In addition, despite the limited coverage of association between *SLC23A1* and UC in existing literatures, we found that *SLC23A1* was significantly downregulated in both UC mice colonic tissues and peripheral blood of UC patients. GSEA revealed that in *SLC23A1*-low UC samples, significantly enriched pathways included the CAMK signaling pathway, electron transfer in mitochondrial complex I, etc. These pathways are closely associated with mitochondrial dysfunction, enhanced oxidative stress, and disturbed energy metabolism. Activation of CAMK signaling can promote inflammatory cytokine release (48, 49). Mitochondrial dysfunction is an important factor in UC pathogenesis (50). Specifically, dysregulation of complex I of the electron transport chain leads to ROS accumulation, which further promotes the opening of the mitochondrial permeability transition pore (mPTP) and subsequently induces cell apoptosis during intestinal inflammation (50, 51). Thus, *SLC23A1* downregulation may exacerbate oxidative stress in UC by compromising mitochondrial function. Immune infiltration analysis showed that *SLC23A1* expression was positively correlated with M2 macrophages, Tregs and CD8 T cells, and negatively correlated with M1 macrophages and neutrophils. Single-cell analysis revealed that *SLC23A1* was differentially expressed in CD4 T cells, naïve B cells, plasma cells, and Tregs. This pattern further indicated that downregulation of this gene may disrupt the protective functions of these cell populations, thereby promoting an M1-skewed and neutrophil-enriched inflammatory environment.

Similar to *SLC23A1*, no published reports have addressed the clinical significance or molecular mechanism of *PHYH* in UC to data. Our GSEA analysis revealed that low *PHYH* expression was enriched in the angiotensin-aldosterone pathway, BMP signaling and the KEAP1_NRF2 signaling pathway, etc. The angiotensin system has been reported to promote colonic inflammation by stimulating Th17 activation (52). Furthermore, dysregulation of BMP signaling has been increasingly implicated in the pathogenesis of IBD (53, 54). Of note, BMP signaling has been linked to neutrophil-driven chronic inflammation (55). Consistent with this, we observed a negative correlation between low *PHYH* expression and neutrophils. This suggested that *PHYH* downregulation may regulate BMP signaling, thereby favoring neutrophil infiltration and chronic inflammation. Moreover, KEAP1_NRF2 signaling pathway enriched in *PHYH* low UC samples have suggested regulation of inflammation and oxidative stress in UC (56). Immune infiltration analysis further showed that *PHYH* expression was positively correlated with M2 macrophages, Tregs, and CD8 T cells, while negatively correlated with M1 macrophages and neutrophils, which closely resembles that of *SLC23A1*. Our single-cell analysis complemented these findings by showing that *PHYH* exhibits broad expression across multiple immune cell types, with particularly high levels in M2 macrophages, plasma cells, CD4 T cells, and CD8 T cells.

Taken together, these findings provide the evidence that *PHYH* downregulation may participate in UC pathogenesis by modulating inflammatory and stress-related signaling pathways and by favoring a pro-inflammatory immune cell composition.

Furthermore, in the single cell analysis, we used the AddModuleScore function to calculate gene scores for each cell type, and the results also showed differences in T-cell and B-cell subsets. These findings enhanced our understanding of the regulatory interplay between the identified BAMGs and immune cells in UC.

While our study identifies key BAMGs associated with UC, several limitations should be acknowledged. First, although machine learning–based analysis successfully prioritized candidate BAMGs, the training set (GSE92415) achieved relatively high AUC values across eight algorithms. However, the external UC tissue datasets also yielded relatively high AUC values, ranging from 0.962 to 1, the datasets based on blood samples showed different performance, ranging from 0.754 to 1. Given the differences in sample sources, GSE119600 (AUC = 0.754) was the reliable indicator of the model’s true generalization ability. Thus, future studies should expand validation to larger, multicenter clinical UC cohorts. Second, while bulk transcriptomic and single-cell RNA sequencing analyses revealed immune expression patterns of the identified BAMGs, functional validation—including loss- or gain-of-function assays in relevant immune cell subsets is required to mechanistically define how these genes modulate immune cell activation, differentiation, or cytokine production. Finally, the three feature genes remain underexplored in clinical value, therefore, our next plan will rigorously explore the associations between these BAMGs and clinical staging, therapeutic response, and prognosis of UC patients.

## Conclusion

In conclusion, our study offers new insights into the role of genes implicated in bile acid metabolism and their association with the development of UC. By integrating machine learning algorithms, we successfully identified three pivotal genes (*CH25H*, *SLC23A1* and *PHYH*) in UC. The three key genes interact with immune cells play an important role in the development of UC, which could be the potential biomarkers.

## Data Availability

The datasets generated and analyzed during the current study are available in the GEO data repository and the accession numbers as follows: GSE92415, GSE87466, GSE87473, GSE107499, GSE126124, GSE119600 and GSE3365. The data used to support the findings of this study are available from the corresponding author upon request.

## Supplementary material

Supplementary Figure 1 Immune cell infiltration analysis.(A) Box plot showed the comparative analysis of the difference in the abundance of immune cell infiltration between UC group and control group. (B) Relative percentage of 22 immune cells in UC samples and controls; (C) Correlation analysis between 11 BAMGs expression and the abundance of immune cell infiltration in UC. Spearman correlation analysis of *SLC23A1* expression and immune cells (D) M1 macrophages; (E) M2 macrophages; (F) neutrophils; (G) monocytes. **P*<0.05, ***P <*0.01, ****P*<0.001.

Supplementary Figure 2 Dot plot showed canonical marker genes used for cell type annotation.

Supplementary Figure 3 Unsupervised cluster analysis based on three feature genes. (A) CDF curve displayed consensus distributions for each k; (B) relative change in the area of the CDF curve; (C) the optimal consensus cluster matrix with k equal to 2; (D) expression patterns of characteristic genes in different clusters; (E) PCA analysis. (F) Comparison of differences in the abundance of immune cell infiltration between C1 and C2. (G) GO enrichment analysis of C1 and C2 by GSVA. (H) KEGG pathways enrichment analysis. **P*<0.05, ***P <*0.01, ****P*<0.001.
