# Supplementary material for: Machine learning–driven identification and experimental validation of key biomarkers in the bile acid metabolic pathway associated with ulcerative colitis
Source: Front Immunol. 2026 Aug 24;17:1873644. doi: 10.3389/fimmu.2026.1873644 (PMC13547527; doi:10.3389/fimmu.2026.1873644)
Supplement: Supplementary file 1 [file Table1.docx]

**Table S1. Datasets obtained from the GEO database in this study.**

| Datasets | Platform | Sample types | UC samples | Control samples |
| --- | --- | --- | --- | --- |
| GSE92415 | GPL13158 | tissue | 162 | 21 |
| GSE87466 | GPL13158 | tissue | 87 | 21 |
| GSE87473 | GPL13158 | tissue | 106 | 21 |
| GSE107499 | GPL15207 | tissue | 75 | 44 |
| GSE126124 | GPL6244 | peripheral blood | 17 | 34 |
| GSE119600 | GPL10558 | peripheral blood | 46 | 47 |
| GSE3365 | GPL96 | peripheral blood | 26 | 42 |
