# Supplementary material for: Machine learning–driven identification and experimental validation of key biomarkers in the bile acid metabolic pathway associated with ulcerative colitis
Source: Front Immunol. 2026 Aug 24;17:1873644. doi: 10.3389/fimmu.2026.1873644 (PMC13547527; doi:10.3389/fimmu.2026.1873644)
Supplement: Supplementary file 2 [file Table2.docx]

**Table S2. Relevant bile acid metabolism genes in this study.**

| SCP2 | HAO1 | ALDH8A1 | GC |
| --- | --- | --- | --- |
| ABCD3 | PEX16 | NR3C2 | ABCA4 |
| SLC27A2 | ABCD1 | ABCA6 | SLC35B2 |
| HSD3B7 | ACSL1 | RETSAT | SLC23A1 |
| HSD17B4 | MLYCD | FADS1 | NUDT12 |
| AKR1D1 | ABCG8 | PECR | ABCA1 |
| ABCD2 | AQP9 | PEX12 | PAOX |
| PEX1 | ISOC1 | CAT | SLC22A18 |
| CYP8B1 | NR1H4 | GNMT | DIO2 |
| PNPLA8 | PEX19 | ABCA9 | RBP1 |
| PHYH | NPC1 | ACSL5 | SLC23A2 |
| SLC27A5 | HSD17B11 | LONP2 | ATXN1 |
| CYP7B1 | AMACR | DHCR24 | GCLM |
| CYP27A1 | CYP46A1 | ABCA8 | NEDD4 |
| PEX26 | PEX13 | PFKM | RXRG |
| AGXT | IDH1 | DIO1 | AR |
| PEX7 | PEX11A | RXRA | BMP6 |
| CROT | HSD17B6 | FDXR | SERPINA6 |
| CYP39A1 | EPHX2 | TTR | LIPE |
| ABCA3 | PRDX5 | KLF1 | EFHC1 |
| CYP7A1 | PXMP2 | SLC29A1 | IDH2 |
| HACL1 | BBOX1 | BCAR3 | IDI1 |
| PIPOX | FADS2 | LCK | CH25H |
| PEX6 | ALDH9A1 | PEX11G | ABCA5 |
| SOD1 | APOA1 | SLCO1A2 | SULT2B1 |
| GNPAT | OPTN | ALDH1A1 | SOAT2 |
| HSD3B1 | GSTK1 | SULT1B1 | TFCP2L1 |
| ABCA2 | ABCG4 | NR1I2 | NR0B2 |
