# Supplementary material for: Machine learning–driven identification and experimental validation of key biomarkers in the bile acid metabolic pathway associated with ulcerative colitis
Source: Front Immunol. 2026 Aug 24;17:1873644. doi: 10.3389/fimmu.2026.1873644 (PMC13547527; doi:10.3389/fimmu.2026.1873644)
Supplement: Supplementary file 3 [file Table3.docx]

**Table S3. Sequences of primers used for RT-qPCR analysis.**

| Species | Genes | Sequences |
| --- | --- | --- |
| Human | β-actin | Forward: 5’- TGTGACGTGGACATCCGCAAAG -3’  Reverse: 5’- TGGAAGGTGGACAGCGAGGC-3’ |
| Human | *SLC23A1* | Forward: 5’GTCCACCTCGTCCAGTCCCA3’  Reverse: 5’ GCACCCGCCCACAAACAT 3’ |
| Human | *PHYH* | Forward: 5’CGGAACAACGGCTGTCTG3’  Reverse: 5’TCGCCCTTCTCCATCACC 3’ |
| Human | *CH25H* | Forward: 5’ CATCCTGTTCTGCCTGCTACTCTT 3’  Reverse: 5’ ACGGAAAGCCAGATGTTGACC 3’ |
