# Supplementary material for: Machine learning–driven identification and experimental validation of key biomarkers in the bile acid metabolic pathway associated with ulcerative colitis
Source: Front Immunol. 2026 Aug 24;17:1873644. doi: 10.3389/fimmu.2026.1873644 (PMC13547527; doi:10.3389/fimmu.2026.1873644)
Supplement: Supplementary file 4 [file Table4.docx]

**Table S4. Clinical characteristics of the UC patients and healthy controls.**

| Variables | HC group (n = 30) | UC group (n = 32) | Statistic  χ²/ Z | *P* |
| --- | --- | --- | --- | --- |
|  |  |  |  |  |
| Gender |  |  |  |  |
| Female/male | 15/15 | 11 /21 | 1.55 | 0.213 |
| Age (years) | 39.00 (34.00, 43.00) | 41.00 (25.75, 54.25) | -0.18 | 0.860 |
| WBC (10^9/L) | 6.02 (4.88, 6.79) | 6.95 (5.54, 9.56) | -2.36 | 0.018 |
| LY (10^9/L) | 1.90 (1.49, 2.34) | 1.73 (1.44, 2.13) | -0.95 | 0.342 |
| MO (10^9/L) | 0.42 (0.32, 0.53) | 0.51 (0.36, 0.78) | -1.82 | 0.069 |
| NE (10^9/L) | 3.49 (2.63, 4.03) | 4.37 (2.78, 6.75) | -2.48 | 0.013 |
| PLT (10^9/L) | 232.50 (195.00, 254.00) | 289.00 (218.00, 381.75) | -2.50 | 0.012 |
| ALT (U/L) | 17.00 (11.25, 28.25) | 15.30 (12.00, 22.25) | -0.90 | 0.367 |
| AST (U/L) | 19.50 (17.00, 28.50) | 18.00 (14.97, 22.75) | -1.59 | 0.111 |
| Urea (mmol/L) | 4.45 (3.92, 5.38) | 4.20 (3.31, 4.92) | -1.31 | 0.190 |
| Cr (μ mol/L) | 66.00 (60.25, 75.00) | 60.55 (49.88, 71.30) | -1.59 | 0.111 |
| TP (g/L) | 73.40 (70.82, 75.57) | 67.40 (61.75, 72.58) | -3.71 | <0.001 |
| ALB (g/L) | 43.10 (42.23, 46.30) | 37.90 (32.77, 43.45) | -3.61 | <0.001 |
| ESR (mm/h) | 8.00 (5.00, 11.00) | 32.50 (10.75, 60.25) | -4.22 | <0.001 |

WBC: white blood cell; LY: lymphocyte; MO: monocyte; NE: neutrophil; PLT: platelet; ALT: alanine aminotransferase; AST: aspartate aminotransferase; Cr: creatinine; TP: total protein; ALB: albumin; ESR: erythrocyte sedimentation rate. HC: healthy control; UC: ulcerative colitis.
